# Predictors of mortality of pediatric burn injury in the Douala General Hospital, Cameroon

**DOI:** 10.11604/pamj.2019.33.189.18498

**Published:** 2019-07-11

**Authors:** Nzozone Henry Fomukong, Alain Chichom Mefire, Gerard Beyiha, Mbuagbaw Lawrence, Mandeng Ma Linwa Edgar, Ngwayu Claude Nkfusai, Samuel Nambile Cumber

**Affiliations:** 1 Department of Medicine and Surgery, Faculty of Health Sciences University of Buea, Buea, Cameroon; 2 Department of Microbiology and Parasitology, Faculty of Science, University of Buea, Buea, Cameroon; 3 Cameroon Baptist Convention Health Services (CBCHS), Yaoundé, Cameroon; 4 Institute of Medicine, Department of Public Health and Community Medicine (EPSO), University of Gothenburg, Box 414, SE-405 30 Gothenburg, Sweden; 5 Faculty of Health Sciences, University of the Free State, Bloemfontein, South Africa; 6 School of Health Systems and Public Health, Faculty of Health Sciences, University of Pretoria Private Bag X323, Gezina, Pretoria, 0001, Pretoria, South Africa

**Keywords:** Pediatric, burns, mortality, predictor

## Abstract

**Introduction:**

Burn injuries are a major cause of hospitalization and are associated with significant morbidity and mortality, particularly in children aged four years or below. In Cameroon, the mortality rate of pediatric severe burns was estimated at 41.2%. There is need to determine the predictors of such mortality in order to guide appropriate management.

**Methods:**

This study is aimed at assessing the predictors of mortality of pediatric patients who sustained a burn injury over a period of 11 years (between 1^st^ of January 2006 and 31^st^ of December 2016) in Douala General Hospital (DGH). The data for this study was entered in an electronic questionnaire and analyzed using Epi info version 7. All variables thought to be associated with mortality were entered in a multiple binary logistic regression model. The magnitude or risk was measured by odds ratio, and the 95% confidence interval was estimated.

**Results:**

A total of 125 cases of pediatric burns were recorded over the study period. A total of 69 (55.65%) were males, giving a male to female ratio of 1.25:1. The median age was 4 years. Most pediatric burns resulted from accidents. Most patient 78 (69%) came before 8 hours following injury. Scalding was the predominant mechanism of injury in 56 (45.5%) of patients. Most patients had partial thickness burn and most burns involved 1-9.9% body surface areas (BSA). The mean length of hospital stay in this study was 7 days, more than half of the patients had no complications during admission. Among those that developed complications, 19 (35%) developed sepsis.

**Conclusion:**

Mortality rate of pediatric burns obtained in this study was 29%, mostly due to cardiac arrest. Flame burns (p=0.03) and BSA >25% (p=0.001) were statistically significant predictors of mortality.

## Introduction

Burn injuries are a major cause of hospitalization and are associated with significant morbidity and mortality, particularly in children aged four years or below [1-3]. Childhood burns place enormous socio-economic burden on individuals, their families and health services [2]. Burns are the fourth commonest type of trauma worldwide, after traffic injuries, falls and interpersonal violence [4]. Nearly one quarter of all burn injuries occur in children under the age of 16 [5], of whom the majority are aged five years and below [5, 6]. Flames, scalds, and contact burns are the top three mechanisms of severe burns in most studies [6-9]. In pediatric populations, scalds represent the most frequent mechanism, accounting for 60% to 75% of all hospitalized burn patients, followed by flame and contact burns [7]. Most patients do not receive pre hospital care, and among patients who receive pre hospital interventions only 17.3% of them receive interventions which are considered to be appropriate (water irrigation) [10]. The main complications noted during the course of admission are wound infections and anaemia [9]. The lack of standardized burn care protocols has a huge impact on African burn care. Some African countries use standard protocols inclusive of the assessment of wound severity [11], administration of analgesia, airway management, intravenous fluids administration, surgery and nutritional support [12]. Other countries have to make do with the expertise and experience of their health care providers. Resuscitation practices follow standard regimes with regard to burn size, resuscitation fluids, monitoring practices, and early feeding [12]. The use of early excision and skin grafting allows initial acute coverage of burns and also reduces necrotic and infected tissue [13]. In addition, early excision and skin grafting leads to decreased lengths of hospital stay, a reduced cost of hospital care, and a significant reduction in mortality [14].

In Cameroon, the mortality rate of pediatric severe burns was estimated at 41.2% [6]. Gender, age, burn size, presence of inhalation injury, presence of comorbidity, and co-existing injury are considered predictors of outcome of burn patients [15-17]. Variations between adult and pediatric patients do exist. Three main reasons for burn mortality common among pediatric patients are identified; burn shock during the first few hours after injury, respiratory failure in the following days, and septic complications and organ failures during the subsequent weeks [18]. Children younger than 48 months with burns involving more than 30% of the body surface have a higher rate of mortality than adults with identical injuries. This is because Children aged less than 48months do not tolerate large thermal injuries as well as adults [19, 20]. The mortality doubles with the development of sepsis along with respiratory failure regardless of total body surface area involved [21]. Acute lung injury and respiratory distress syndrome (ARDS) account for 40-50% of all deaths. Multi-drug resistant organisms also increase death rates in patients with burn-related sepsis from 42% to 86% [22]. Delay of resuscitation is another very important predictor of death, [22] which has been measured as length of time to intravenous access. Patients receiving resuscitation within the first hour have significantly higher chances of survival [23], total body surface area (TBSA) >36% is found to be associated with the highest mortality [24]. This study aimed at identifying the predictors of outcome of pediatric burns in the DGH. This study will provide surgeons, clinicians and other health practitioner's information on factors to consider in the management of burns in children.

## Methods

**Study design:** this study was a retrospective cross-sectional observational study with review of files over a period of 11 years (from January 1^st^ 2006 to December 31^st^ 2016) of patients admitted in DGH burns intensive care unit.

**Study area and setting:** the study was carried out in the DGH, located in Douala at Beedi, next to Malangue, latitude 4º3”55.08”, longitude 9º45”31.32”. Douala is the economic capital of Cameroon and has a population of about 2,865,795 individuals [25]. The DGH is the only hospital with a tertiary Burn Intensive Care Unit in the Littoral region, enabling patient referrals from other hospitals and clinics in the region, as well as peripheral regions like south west and west regions. The hospital covers a length of 1.26 kilometres. The burn intensive care unit is located on the ground flow and has its own emergency unit, dressing room and drugs store. Entry is restricted with an alarm door. Visiting hours are also restricted. Hand washing and scrubbing is obligatory for all visitors and none intensive care unit staffs. The burn intensive care unit has a capacity of more than 25 beds and a good record keeping platform. Physiotherapy and imaging is done in the burn unit to limit patient exit.

**Study population and sample:** the study population was made up of all pediatric burns patient records admitted in DGH from 1^st^ of January 2006 to 31^st^ December 2016.

**Inclusion criteria:** all admitted burns patient records with complete demographic data within study period. Records of patient age 0-16 with complete documentation of mechanism of burn, clinical profile including TBSA involved, treatment modalities, outcome, cause of adverse outcome.

**Exclusion criteria:** individuals brought in dead, patients who came for check-up following electrocution without incurring burns and patients managed as outpatients.

**Study procedure:** ethical clearance was sort and granted by the Faculty of Health Sciences Ethics Review Board, after submission of a copy of research protocol. Administrative clearance was obtained from the Medical Administration of the DGH. Once the approvals were obtained, patient records and folders in the DGH burn intensive care unit within the study period was obtained and reviewed for completeness. Complete records were entered into a data collection sheet. The record and files were returned to the burn centre archive after the data was collected.

**Data management:** the data that was collected and the paper checklist was entered into an electronic questionnaire created in Epi info by the researcher. The electronic data was saved in a folder in a computer that was accessible only to the researcher and the hard paper questions were locked in a cupboard that was also accessible only to the researcher.

**Data analysis:** the collected data was analyzed using the statistical software program Epi info version 7. The burns and socio-demographic characteristics of patients were described using frequencies and percentages for categorical variables and medians and interquartile ranges for continuous variables. To assess the predictors of death resulting from burns, bivariate and multivariate analysis were done on the age of patients, sex, mechanism of burns, complications, initial treatment at site of injury, time taken to arrive the hospital following the occurrence of burns, burn depth and burn size. The bivariate analysis comprised considering deaths from burns as a binary outcome variable and burns/socio-demographic characteristics of patients as predictors and then computing the odds of death resulting from burns. Unadjusted odds ratios, 95% confidence intervals and p-values were computed. A p-value ≤0.25 was set as the determining point for a variable to be considered as appearing to have an association with death from burns and to be included in the multivariate logistic model [15]. For the multivariate logistic regression analysis, death from burns was considered as a binary outcome variable and burns/sociodemographic variables of patients that had p-values ≤0.25 in the bivariate analysis as predictors. Adjusted odds ratios, 95% confidence intervals and p-values were computed. All variables with p-values <0.05 were considered as having a statistical significant association with death from burns. The results were presented in tables and charts followed by discussion in chapter four and five.

**Ethical consideration:** ethical clearance was sort and granted by the Faculty of Health Sciences Ethics Review Board, after submission of a copy of research protocol. The research was carried out after the administrative and ethical approvals were obtained. The data was collected and entered only by the researcher to ensure confidentiality and data collection kept in a password protected folder in a computer assessable only by the researcher.

**Limitations:** this study entailed going into hospital files, some data were not present such as level of education of the care givers, occupation of parents, topical treatment and antiseptics used for wound dressing. Since we relied only on what was recorded in the files, complications and outcome following discharge could not be assessed.

## Results

The number of admitted pediatric cases (0-16 years) within the study period was 125 (28.03%), with a male predominance of 69 (55.65%) giving a male to female ratio of about 1.25:1. The socio-demographic/burn correlates of patients' death in relation to burns managed at the DGH. In the bivariable analysis, the factors that appeared to be associated with patients' death resulting from burns included complications of burns, initial treatment given to a patient with burns, burn depth, burn size and mechanism of burns (Table 1). However, after adjusting for potential confounding by each of the socio-demographic/burn factors that appeared to have an association with death from burns in the bivariate analysis, only having burn sizes greater than 25% and having burns resulting from electrical and flame mechanisms remained significant predictors of death from burns. Actually, the odds of death from burns in participants with burn sizes greater than 25% was 30.1 times (95%CI: 6.54, 80.96) that in participants with burn sizes of 25% or less. Finally, the odds of death from burns comparing participants with flame mechanisms of burns to participants with scalds, chemical, electrical and other mechanisms of burns was 7.26 times (95%CI: 1.95, 27.04) (Table 2).

**Table 1:** Correlates of pediatric burns death-bivariable analysis

Characteristic	N	%	Patient death from burns		P-values
			OR*	95% CI	
**Age**					
0-5	23	32.9	Ref	-	-
>5	13	24.1	0.65	0.29-1.44	0.29
**Sex**					
Male	22	31.9	Ref	-	-
Female	13	24.1	0.68	0.30-1.51	0.34
**Complication of burns**					
Shock	23	92.7	Ref	-	-
All other complications	13	21.1	0.02	0.003-0.17	0.001
**Initial treatment at burn site**					
Yes	1	7.7	Ref		
No	35	31.5	5.53	0.69-44.19	0.06
**Burn depth**					
Superficial	90	35.6	Ref		
Deep	32	23.4	0.5	0.25-1.22	0.14
**Burn size**					
0-25%	4	5.2	Ref	-	-
>25%	32	68.1	38.9	11.9-126.5	0.01
**Mechanism of burn**					
Scald, chemical, Electrical and others	14	16.8	Ref		
flame	21	56.3	6,75	2 .84-16.05	0.001
**Time to hospital following burns**					
0-8 hours	23	28.8	Ref		
>8 hours	9	36.0	1.39	0.4-3.60	0.49

* OR=unadjusted odds ratio, OR=odds ratio, Ref= reference variable category, CI=confidence interval, P-values<0.25 suggests possible association to death from burns.

**Table 2:** correlates of pediatric burns death- multivariable analysis

Characteristic	N	%	Patient death from burns		P-values
			aOR*	95% CI	
**Complication of burns**					
Shock	23	92.7	Ref	-	-
All other complications	13	21.1	0.14	0.02-1.29	0.08
**Initial treatment at burn site**					
Yes	1	7.7	Ref		
No	35	31.5	2.28	0.20-25.55	0.50
**Burn depth**					
Superficial	90	35.6	Ref		
Deep	32	23.4	0.53	0.17-1.72	0.29
**Burn size**					
0-25%	4	5.2	Ref	-	-
>25%	32	68.1	30.1	6.54-80.96	0.001
**Mechanism of burn**					
Scald, chemical, Electrical, others	14	16.8	Ref		
flame	21	56.3	7.26	1.95-27.04	0.03

* aOR=adjusted odds ratio, OR=odds ratio, Ref= reference variable category, CI=confidence interval, P-values<0.05 are statistically significant

## Discussion

The proportion of pediatric burns (0-16 years) hospitalized in the Douala General Hospital during this study was 125 (28.03%). There was a male predominance of 69 (55.65%) similar to results in a study conducted in Yaoundé (Cameroon) [6]. Flames, scalds, and electrical burns were the top three mechanisms of severe burns in this study. This was similar to other studies carried out in Yaoundé Central Hospital, Europe, Bordeaux, Nigeria, Ghana, South Africa and India. [6-9, 15, 17]. Overall in this study, most of the burns (45.5%) resulted from scalds.

In Cameroon, Amengle *et al.* estimated the mortality rate of pediatric severe burns at 41.2% [6]. In this study, 32 patients died in the course of treatment giving a mortality rate of 29%. Most (60.5%) of pediatric burns patients received in the DGH were treated and discharged. the mortality rate obtained in this study was lower than that obtained by Amengle *et al.* but similar to the results obtained in Ghana by Agbenorku *et al.* [24] and India by Mukerji *et al.* [21]. In most of the patients (41.7%), the cause of death was not recorded. Gender, age, burn size, delay of resuscitation, presence of inhalation injury, presence of complications in the course of treatment, presence of comorbidity, and co-existing injury are considered predictors of outcome of burn patients [15-17]. In this study, after adjusting for potential confounding by each of the socio-demographic/burn factors that appeared to have an association with death from burns in the bivariate analysis, only having burn sizes greater than 25% (p=0.001) and having burns resulting from flame mechanisms (p=0.03) remained significant predictors of death from burns. The odds of death from burns in participants with burn sizes greater than 25% was 30.1 times (95%CI: 6.54, 80.96) that in participants with burn sizes of 25% or less.

Finally, the odds of death from burns comparing participants with flame mechanisms of burns to participants with scalds, electrical, chemical and other mechanisms of burns was 7.26 times (95%CI: 1.95, 27.04). In this study, gender, age, burn depth, prehospital interventions, delay of resuscitation proved not to be predictors of mortality in pediatric burns. These results were different from those obtained in Ghana by Agbenorku *et al.* [24]. In the study conducted by Agbenorku *et al.* predictors of outcome identified were Age <6 years (P=0.028), Scald especially hot water and soup (P=0.016), TBSA >36% (P=0.028) and inhalation injury (P=0.040). In the study conducted by Sharma *et al.* predictors of outcome were age <5years, flame burns, BSA >70%. Predictors of outcome common to all studies is BSA, and mechanisms. BSA affects mortality mainly because, the more the surface area burned, the more fluids are lost, the more liable the patient is to infections and multiple organ shut down. In other countries, the odds of death start increasing when the burn size is >36%. But in our setting, it was as low as 25% (23 times risk of dead).

## Conclusion

This study was aimed at analyzing the epidemiological profile, mechanisms and predictors of outcome of pediatric burns so as to reduce incidence, morbidity and mortality. Most burn injuries were partial thickness and burn size was 0-9.9%. The most affected body regions were the lower limbs, trunk and upper limbs respectively. The mortality rate was 29% mostly from cardiac arrest. Predictors of outcome in this study were burn size >25%, flame burns.

### What is known about this topic

- In other countries, the odds of death starts increasing when the burn size is >36%. Study done in Ghana [15];
- In Cameroon, the mortality rate of pediatric severe burns was estimated at 41.2% [6];
- Gender, age, burn size, presence of inhalation injury, presence of comorbidity, and co-existing injury are considered predictors of outcome of burn patients [15-17].

### What this study adds

- This is the first study to the best of our knowledge and literature search done in Cameroon to assess predictors of dead of pediatric burns in Cameroon;
- In our setting, the odds of death starts increasing when the burn size is as low as 25% (23 times risk of dead). People die more in Cameroon from burns with lower body surface area affected than in other developing countries;
- Mortality rate of pediatric burns was lower in this study (29%) compared to another study done in Cameroon 41.2%, in this study only flame and burn size were statistically significant predictors of mortality.

## Competing interests

The authors declare no competing interests.
